# Temporal Associations Among Body Mass Index, Fasting Insulin, and Systemic Inflammation: A Systematic Review and Meta-analysis

**DOI:** 10.1001/jamanetworkopen.2021.1263

**Published:** 2021-03-12

**Authors:** Natasha Wiebe, Feng Ye, Ellen T. Crumley, Aminu Bello, Peter Stenvinkel, Marcello Tonelli

**Affiliations:** 1 Department of Medicine, University of Alberta, Edmonton, Alberta, Canada; 2 Department of Health, St Francis Xavier University, Antigonish, Nova Scotia, Canada; 3 Department of Renal Medicine M99, Karolinska University Hospital, Stockholm, Sweden; 4 Department of Medicine, University of Calgary, Calgary, Alberta, Canada

## Abstract

**Importance:**

Obesity is associated with a number of noncommunicable chronic diseases and is purported to cause premature death.

**Objective:**

To summarize evidence on the temporality of the association between higher body mass index (BMI) and 2 potential mediators: chronic inflammation and hyperinsulinemia.

**Data Sources:**

MEDLINE (1946 to August 20, 2019) and Embase (from 1974 to August 19, 2019) were searched, although only studies published in 2018 were included because of a high volume of results. The data analysis was conducted between January 2020 and October 2020.

**Study Selection and Measures:**

Longitudinal studies and randomized clinical trials that measured fasting insulin level and/or an inflammation marker and BMI with at least 3 commensurate time points were selected.

**Data Extraction and Synthesis:**

Slopes of these markers were calculated between time points and standardized. Standardized slopes were meta-regressed in later periods (period 2) with standardized slopes in earlier periods (period 1). Evidence-based items potentially indicating risk of bias were assessed.

**Results:**

Of 1865 records, 60 eligible studies with 112 cohorts of 5603 participants were identified. Most standardized slopes were negative, meaning that participants in most studies experienced decreases in BMI, fasting insulin level, and C-reactive protein level. The association between period 1 fasting insulin level and period 2 BMI was positive and significant (β = 0.26; 95% CI, 0.13-0.38; *I*^2^ = 79%): for every unit of SD change in period 1 insulin level, there was an ensuing associated change in 0.26 units of SD in period 2 BMI. The association of period 1 fasting insulin level with period 2 BMI remained significant when period 1 C-reactive protein level was added to the model (β = 0.57; 95% CI, 0.27-0.86). In this bivariable model, period 1 C-reactive protein level was not significantly associated with period 2 BMI (β = –0.07; 95% CI, –0.42 to 0.29; *I*^2^ = 81%).

**Conclusions and Relevance:**

In this meta-analysis, the finding of temporal sequencing (in which changes in fasting insulin level precede changes in weight) is not consistent with the assertion that obesity causes noncommunicable chronic diseases and premature death by increasing levels of fasting insulin.

## Key Points

**Question:**

What are the temporal associations among higher body mass index (BMI) and chronic inflammation and/or hyperinsulinemia?

**Findings:**

In this systematic review and meta-analysis of 5603 participants in 112 cohorts from 60 studies, the association between period 1 (preceding) levels of fasting insulin and period 2 (subsequent) BMI was positive and significant: for every unit of SD change in period 1 insulin level, there was an ensuing associated change in 0.26 units of SD in period 2 BMI.

**Meaning:**

These findings suggest that adverse consequences currently attributed to obesity could be attributed to hyperinsulinemia (or another proximate factor).

## Teaser

This systematic review and meta-analysis summarize evidence on the temporality of the association between higher body mass index and chronic inflammation and/or hyperinsulinemia.

## Introduction

Obesity is associated with a number of noncommunicable chronic diseases (NCDs), such as type 2 diabetes, coronary disease, chronic kidney disease, and asthma. Although obesity is also purported to cause premature death, this association fails to meet several of the Bradford Hill criteria for causation.^1,2^ First, the putative attributable risk of death is small (<5%).^3^ Second, the dose-response gradient between body mass index (BMI) and mortality is U-shaped with overweight (and possibly obesity level I) as the minima.^3^ Third, evidence from animal models comes largely from mice that have been fed high-fat diets; unlike humans, these animals did not normally have fat as part of their typical diet, and thus the experiments are potentially not analogous to those in humans. Fourth, evidence that people with obesity live longer than their lean counterparts in populations with acute or chronic conditions and older age is remarkably consistent.^4,5,6,7,8,9,10,11,12,13,14,15,16^ Therefore, it is possible that rather than being a risk factor for NCDs, obesity is actually a protective response to the development of disease.

The putative links between obesity and adverse outcomes are often attributed to 2 potential mediators: chronic inflammation and hyperinsulinemia. These characteristics have been associated with several NCDs, including obesity as well as type 2 diabetes, cardiovascular disease,^17^ and chronic kidney disease.^18^ Existing data on the association of obesity with chronic inflammation and/or hyperinsulinemia are chiefly cross-sectional, making it difficult to confirm the direction of any causality. This systematic review and meta-analysis summarizes evidence on the temporality of the association between higher BMI and chronic inflammation and/or hyperinsulinemia. We hypothesized that changes in chronic inflammation and hyperinsulinemia would precede changes in higher BMI.

## Methods

This systematic review and meta-analysis was conducted and reported according to Preferred Reporting Items for Systematic Reviews and Meta-analyses (PRISMA)^19^ and Meta-analysis of Observational Studies in Epidemiology (MOOSE)^20^ reporting guidelines. Research ethics board approval was not required because this is a systematic review of previously published research.

### Data Sources and Searches

We performed a comprehensive search designed by a trained librarian (E.T.C.) to identify all longitudinal studies and randomized clinical trials (RCTs) that measured fasting insulin and/or an inflammation marker and weight with at least 3 commensurate time points. We included only primary studies published in the English language as full peer-reviewed articles. MEDLINE (1946 to August 20, 2019) and Embase (1974 to August 19, 2019) were searched; however, only studies published in 2018 were retained because of the high volume of results. No existing systematic reviews were found. The specific search strategies are provided in eTable 1 in the Supplement. The abstracts were independently screened by 2 reviewers (including N.W.). The full text of any study considered potentially relevant by 1 or both reviewers was retrieved for further consideration. The data analysis was conducted between January 2020 and October 2020.

### Study Selection

Each potentially relevant study was independently assessed by 2 reviewers (N.W. and F.Y.) for inclusion in the review using the following predetermined eligibility criteria. Longitudinal studies and RCTs with men and nonpregnant and not recently pregnant women (≥18 years of age) and at least 3 time points with 1 or more weeks of follow-up in which fasting insulin levels or a marker of inflammation and some measure of weight were included in this review. Disagreements were resolved by consultation.

### Data Extraction and Risk of Bias Assessment

Data from eligible studies were extracted by a single reviewer (N.W.). A second reviewer checked the extracted data for accuracy. The following properties of each study were recorded in a database: study characteristics (country, era of accrual, design, duration of follow-up, populations of interest, intervention where applicable, and sample size), age and sex of participants, and the measures of interest (numbers, means, and SDs) for all time points: (1) fasting insulin, the homeostatic model assessment index, or the quantitative insulin sensitivity check index; (2) concentrations of C-reactive protein (CRP), interleukin cytokines, or tumor necrosis factor; and (3) weight, BMI (calculated as weight in kilograms divided by height in meters squared), fat mass, or fat mass percentage.

Risk of bias was assessed using items from Downs and Black^21^: clear objective, adequate description of measures, sample size or power calculation, intention to treat study design (in those studies that assigned the intervention), adequate description of withdrawals, adequate handling of missing data, and adequate description of results. Source of funding was also extracted, given its potential to introduce bias.^22^

### Statistical Analysis

Data were analyzed using Stata software, version 15.1 (StataCorp LLC). Missing SDs were imputed using interquartile ranges or using another SD from the same cohort.^23^ Data were extracted from graphs if required.

To determine a likely temporal sequencing of fasting insulin level or chronic inflammation with obesity, we compared the associations of period 2 insulin level or inflammation regressed on period 1 BMI and period 2 BMI regressed on period 1 insulin or inflammation. A stronger association would support a particular direction of effect.

For each measure of interest, the change in means was calculated between adjacent time points and divided by the number of weeks between the measures. This slope or per week change in measure was then standardized by dividing it by the pooled SD, giving a standardized slope. Because of expected diversity among studies, we decided a priori to combine the standardized slopes using a random-effects models. Period 2 standardized slopes of weight measures were regressed onto period 1 standardized slopes of insulin or inflammation measures and vice versa. We regressed measures of insulin post hoc on measures of inflammation and vice versa.

The type I error rate for meta-regressions was set at a 2-sided *P* < .05. Statistical heterogeneity was quantified using the τ^2^ statistic (between-study variance)^24^ and the *I*^2^ statistic. Differences in standardized slopes (βs) along with 95% CIs are reported.

We considered a number of sensitivity analyses. Because we included multiple standardized slopes at different intervals from the same studies (or same cohorts), we accounted for this nonindependence using a generalized linear model in which the family was gaussian and the link was identity, which allowed for nested random effects (results by intervals were nested within cohorts). To estimate between-study heterogeneity, the coefficients for the within-cohort SEs were constrained to 1. We also performed 2 subgroup analyses: whether the study population had undergone bariatric surgery and the numbers of weeks between time points (>12 vs ≤12 weeks), reasoning that if the effects of one measure of interest acted quickly on the other, then shorter intervals might demonstrate stronger associations. We explored post hoc models with 2 measures of interest as period 1 independent variables.

## Results

### Quantity of Research Available

**Figure 1.**
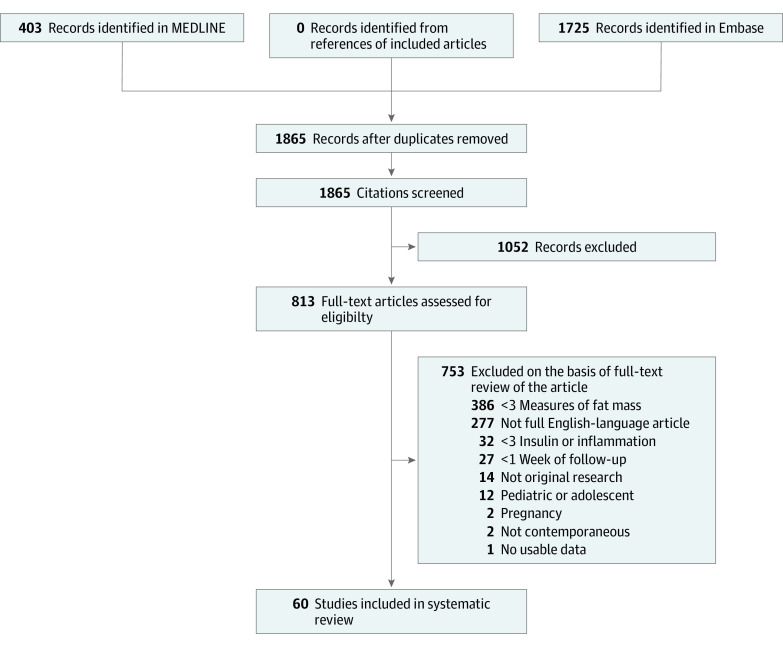
Flow Diagram of Studies

The searches identified 1865 unique records identifying articles or abstracts published in 2018 (Figure 1). After the initial screening, the full texts of 813 articles were retrieved for detailed evaluation. Of these, 753 articles were excluded, resulting in 60 that met the selection criteria and 5603 enrolled participants (of whom 5261 were analyzed).^25,26,27,28,29,30,31,32,33,34,35,36,37,38,39,40,41,42,43,44,45,46,47,48,49,50,51,52,53,54,55,56,57,58,59,60,61,62,63,64,65,66,67,68,69,70,71,72,73,74,75,76,77,78,79,80,81,82,83,84^ We decided to exclude 12 studies of children and adolescents post hoc because these studies used different BMI measures. Disagreements about the inclusion of studies occurred in 2% of the articles (κ = 0.87).

### Characteristics of Studies

There were 26 RCTs, 4 nonrandomized clinical trials, 23 prospective cohort studies (3 nested within an RCT), and 7 retrospective cohort studies (Table 1^25,26,27,28,29,30,31,32,33,34,35,36,37,38,39,40,41,42,43,44,45,46,47,48,49,50,51,52,53,54,55,56,57,58,59,60,61,62,63,64,65,66,67,68,69,70,71,72,73,74,75,76,77,78,79,80,81,82,83,84^). Of the studies, 58% began data collection in the 5 years before publication. The earliest study accrued participants starting in 2000. The durations of follow-up ranged from 1 to 60 months (median, 12 months). A total of 21 studies were from Western Europe,^25,27,41,54,61,62,65,72,81,82,84^ 11 from North America,^25,27,41,54,61,62,65,72,81,82,84^ 9 from East Asia,^29,38,39,44,51,52,60,66,68^ 5 from South America,^28,69,78,79,83^ 5 from Western Asia,^28,69,78,79,83^ and 3 each from Africa,^35,50,76^ the Pacific,^26,42,75^ and Eastern Europe.^45,74,80^

**Table 1.** Study and Study Population Characteristics

Source	Country	Year of study start	Design	Follow-up, mo	Population	Cohort(s)	Enrolled/analyzed	Mean age, y	Male, %
Abdel-Razik et al,^35^ 2018	Egypt	2015	RCT	6	NASH	Rifaximin and placebo	25 and 25	40 and 38	36 and 28
Abiad et al,^37^ 2018	Lebanon	2015	Prospective cohort	12	BMI ≥40 or >35 with a comorbidity and SG	PCOS and control	6 and 19/16	24 and 28	0
Arikawa et al,^82^ 2018	US	2009	RCT	4	BMI ≥27 after breast cancer	CR diet plus exercise and weight management counseling	10/10 and 11/10	55 and 58	0
Arnold et al,^72^ 2018	US	2015	Prospective cohort	3	BMI >30	Decreased added sugars, increased fiber, and fish diet	15/14	59	0
Asle Mohammadi Zadeh et al,^55^ 2018	Iran	NR	RCT	6	T2D	Low-carbohydrate diet, low-fat diet, high-fat diet, and control	11, 11, 11, and 9	47, 49, 45, and 45	100
Baltieri et al,^83^ 2018	Brazil	2015	Prospective cohort	12	BMI ≥35	RYGB	19/13	37^a^	0
Bulatova et al,^46^ 2018	Jordan	2012	RCT	6	Prediabetes or T2D	Metformin and control	42/26 and 49/27	51 and 51	22 and 3
Carbone et al,^67^ 2019^b^	Italy	2007	Retrospective cohort	36	RYGB or BPD with T2D	T2D remission and no T2D remission	14 and 27	54^a^ and 56^a^	64 and 82
Chen et al,^29^ 2018	China	2012	nRCT	12	T2D	Saxagliptin and metformin and acarbose and metformin	51 and 51	64 and 64	47 and 47
Cheung et al,^42^ 2018	Australia	NR	Prospective cohort	36	Prostate cancer	Cessation of androgen deprivation therapy and control	34/27 and 29/19	68^a^ and 71^a^	100
Chiappetta et al,^73^ 2018	Germany	2014	Retrospective cohort	6	BMI ≥40 or ≥35 with a comorbidity	SG, 1-anastomosis GB, and RYGB	241, 68, and 159	44	32
Dardzińska et al,^74^ 2018	Poland	NR	Prospective cohort	12	BMI >35 with no diabetes medication and no cardiovascular events	Mini-GB, SG, and RYGB	12/9, 8/5, and 11/9	38	24
De Luis, Calvo et al,^34^ 2018	Spain	NR	Prospective cohort	36	BMI ≥40 or >35 with a comorbidity after bariatric surgery	CC^c^ rs266729 CG and GG rs266729	84 and 65	47 and 47	23 and 25
De Luis, Izaola et al,^49^ 2018	Spain	NR	Prospective cohort	36	BMI ≥30	Mediterranean CR diet then dietary counseling	335	50	25
De Luis, Pacheco et al,^64^ 2018	Spain	NR	Prospective cohort	36	BPD with no diabetes and BMI ≥40	GG^c^ rs670 and GA or AA rs670	46 and 17	48 and 47	13 and 18
De Paulo et al,^28^ 2018	Brazil	2015	RCT	8	Aromatase inhibitor after breast cancer	Aerobic and resistance training and stretching and relaxation exercises	18 and 18	63 and 67	0
Demerdash et al,^76^ 2018	Egypt	2011	Prospective cohort	24	Obesity	SG	92	43	30
Derosa et al,^40^ 2018	Italy	NR	RCT	12	T2D and hypertension	Canrenone and hydrochlorothiazide	92 and 90	53 and 53	52 and 49
Dhillon et al,^84^ 2018	US	2016	RCT	2	College students (no diabetes or prediabetes)	Almond snacks and cracker snacks	38 and 35	18 and 18	42 and 46
Di Sebastiano et al,^27^ 2018	Canada	NR	Prospective cohort	8	Prostate cancer	Treated	9	71	100
Drummen et al,^59^ 2018 (PREVIEW)	Netherlands	2013	Prospective cohort nested in RCT	24	BMI >25 and prediabetes	High-protein diet and moderate-protein diet	12 and 13	58 and 54	50 and 58
Esquivel et al,^69^ 2018	Argentina	2009	Prospective cohort	12	BMI >40 or >35 with a comorbidity	SG	63/43	40	35
Fortin et al,^54^ 2018	Canada	2016	RCT	9	T1D and metabolic syndrome	Mediterranean diet and low-fat diet	14 and 14	52 and 50	47 and 64
Fuller et al,^26^ 2018 (DIABEGG)	Australia	2013	RCT	6	Prediabetes or T2D	High-egg diet and low-egg diet	72/66 and 68/62	60 and 61	50 and 42
Gadéa et al,^56^ 2018	France	2011	Prospective cohort	6	Breast cancer	Chemotherapy	52	60^a^	0
Galbreath et al,^62^ 2018	US	NR	RCT	3	BMI >27 or body fat >35%	High-protein diet, high-carbohydrate diet, and control	24/17, 24/18, and 24/19	66, 63, and 66	0
Goday et al,^77^ 2018	Spain	2010	Retrospective cohort	24	SG and RYGB	*Helicobacter pylori* eradication and control for SG and *H pylori* and eradication control for RYGB	49 and 60 (SG) 50 and 70 (RYGB)	44 and 46 (SG) and 42 and 42 (RYGB)	37 and 22 (SG) and 22 and 16 (RYGB)
Guarnotta et al,^36^ 2018	Italy	2013	Prospective cohort	12	Cushing disease	Pasireotide	12	40^a^	17
Hady et al,^45^ 2018	Poland	2012	RCT	12	Obesity	32F bougie size in SG, 36F bougie size in SG, and 40F bougie size in SG	40, 40, and 40	41, 43, and 45	38, 30, and 43
Hanai et al,^44^ 2018	Japan	NR	RCT	1	Surgery for head and neck squamous cell carcinoma	EPA-enriched nutritional supplement and control	13 and 14	62 and 66	62 and 57
Hattori,^66^ 2018	Japan	2016	RCT	12	SGLT2 inhibitors in T2D	Empagliflozin and placebo	51 and 51	57 and 58	75 and 80
Kazemi et al,^61^ 2018	Canada	2011	RCT	12	PCOS	Low-glycemic index pulse-based diet and therapeutic lifestyle change diet	47/31 and 48/30	27 and 27	0
Keinänen et al,^53^ 2018	Finland	2010	Prospective cohort	12	First-episode psychosis	Treated	95	25^a^	68
Krishnan et al,^25^ 2018	US	2015	RCT	2	BMI of 25-39.9	2010 American dietary guidelines diet and typical American diet	28/22 and 24/22	47 and 47	0
Lambert et al,^79^ 2018	Brazil	NR	Retrospective cohort	12	BMI >40 or BMI >35 with comorbidity or BMI >30 and T2D	RYGB and BPD	108	44	42
Lee et al,^38^ 2018	Singapore	2009	Retrospective cohort	36	Prediabetes	Bariatric surgery and control	44 and 25	43 and 50	34 and 12
Liang et al,^52^ 2018	Taiwan	2008	Prospective cohort	60	WC ≥90, MetS, no diabetes	Low-calorie diet	40/18	46	100
Liaskos et al,^70^ 2018	Greece	NR	nRCT	6	BMI >40 and no T2D	SG and RYGB	43 and 28	38 and 38	21 and 25
Liu et al,^60^ 2018	China	2014	Retrospective cohort	12	T2D and BMI ≥28	RYGB	45	44	100
Madjd et al,^33^ 2018	Iran	2014	RCT	18	BMI of 27-40	Diet beverages and water	36 and 35	32 and 32	0
Most et al,^41^ 2018 (CALERIE 2)	US	2007	Prospective cohort nested in RCT	24	BMI of 22-27.9 plus ≥5% weight loss in CR 25% and <5% weight loss in ad libitum	25% CR and control	47/34 and 26/19	40 and 39	29 and 37
Mraović et al,^80^ 2018	Serbia	2014	RCT	10	BMI ≥35	20% CR diet, 50% CR diet, and alternating 70% and 30% CR diet	37, 30, and 30	31, 32, and 32	0
Munukka et al,^71^ 2018	Finland	NR	Prospective cohort	1	BMI >27.5 with no major comorbidity	American College of Sports Medicine exercise program	19/17	37	0
Nicoletto et al,^78^ 2018	Brazil	2014	Prospective cohort	12	CKD	Kidney transplantation	46	49	59
Nilholm et al,^30^ 2018	Sweden	2014	Prospective cohort	6	T2D	Okinawan-based Nordic diet	30	58	43
Nishino et al,^39^ 2018	Japan	2011	RCT	1	Esophagectomy for esophageal cancer	Daikenchuto (TJ-100) and control	19 and 20	68^a^ and 61^a^	89 and 80
Patel et al,^57^ 2018	United Kingdom	NR	nRCT	18	BMI of 30-50 and T2D	Duodenal-jejunal sleeve bypass	45	50	49
Rajan-Khan et al,^81^ 2018	US	2011	RCT	4	BMI ≥25	Mindfulness-based stress reduction and health education	42 and 44	47 and 42	0
Rubio-Almanza et al,^32^ 2018	Spain	2000	Retrospective cohort	60	Prediabetes or T2D and bariatric surgery	Prediabetes and T2D	57/38 and 48/32	48	17
Schübel et al,^58^ 2018 (HELENA)	Germany	2015	RCT	12	BMI of 25-39.9	5:2 intermittent CR diet, continuous CR diet, and control	49, 49, and 52	49, 51, and 51	51, 51, and 48
Shah et al,^65^ 2018 (EVADE CAD)	US	2014	RCT	2	Coronary artery disease	Vegan diet and AHA diet	50 and 50	63^a^ and 60^a^	86 and 84
Sherf-Dagan et al,^31^ 2018	Israel	2014	RCT	12	NAFLD after SG	Probiotic and placebo	50/40 and 50/40	42 and 44	40 and 45
Stolberg et al,^48^ 2018	Denmark	2012	RCT	24	RYGB	Moderate-intensity physical training and control	32 and 28	43 and 43	34 and 25
van Dammen et al,^43^ 2018	Netherlands	2009	RCT	6	BMI ≥29 and infertily	Lifestyle intervention and control	290/289 and 287/285	30 and 30	0
van Rijn et al,^47^ 2018	Netherlands	2014	Prospective cohort nested in RCT	12	BMI of 30-50 and T2D	Duodenal-jejunal bypass liner	28	50^a^	39
Wilson et al,^50^ 2018	South Africa	2005	Prospective cohort	2	TB symptoms	Treated for TB and control	295 and 93	34^a^	56
Witczak et al,^63^ 2018	United Kingdom	NR	Prospective cohort	6	BMI >40 and T2D or IGT	Bariatric surgery	20	51	35
Wormgoor et al,^75^ 2018	New Zealand	2015	RCT	9	T2D	HIIT and moderate-intensity continuous training	12/11 and 11	52 and 53	100
Yang et al,^68^ 2018	China	2015	nRCT	12	BMI >35 or ≥30 with a comorbidity	SG and RYGB	32/10 and 28/10	32 and 32	50 and 50
Zhang et al,^51^ 2018	China	2015	RCT	6	Dyslipidemia	Coenzyme Q10 and placebo	51 and 50	52 and 50	28 and 36

Abbreviations: AHA, American Heart Association; BMI, body mass index (calculated as weight in kilograms divided by height in meters squared); BPD, biliopancreatic diversion surgery; CKD, chronic kidney disease; CR, calorie restriction; EPA, eicosapentaenoic acid; GB, gastric bypass; HIIT, high-intensity interval training; IGT, intolerance glucose test; MetS, metabolic syndrome; NAFLD, nonalcoholic fatty liver disease; NASH, nonalcoholic steatohepatitis; NR, not reported; nRCT, nonrandomized clinical trial; PCOS, polycystic ovary syndrome; RCT, randomized clinical trial; RYGB, Roux-en-Y gastric bypass; SG, sleeve gastrectomy; SGLT2, sodium-glucose transport protein 2; T1D, type 1 diabetes; T2D, type 2 diabetes; TB, tuberculosis; WC, waist circumference.

^a^ Median.

^b^ Published online in 2018.

^c^ AA, CC, CG, GA, and GG are alleles.

A total of 90% of the studies were in populations with metabolic disease or conditions associated with metabolic disease: obesity,^25,32,33,34,37,41,43,45,47,48,49,52,57,58,59,60,62,63,64,67,68,69,70,71,72,73,74,76,77,79,80,81,82,83^ diabetes or prediabetes,^26,32,38,46,59^ hypertension,^40^ coronary artery disease,^65^ dyslipidemia,^51^ chronic kidney disease,^78^ nonalcoholic fatty liver disease,^31,35^ Cushing disease,^36^ polycystic ovary syndrome,^61^ breast cancer,^28,56,82^ and aging (ie, college students^84^). Of the patients in these 54 studies, 22 (41%) had undergone bariatric surgery as the studied intervention (n = 14) or as part of the required eligibility criteria (n = 8). Other populations were subjected to operations or therapies that adversely cause lean mass loss and/or fat mass gain, such as prostate,^27,42^ esophageal,^39^ head and neck squamous cell^44^ cancers, and psychosis,^53^ or where the disease course itself (ie, tuberculosis) causes lean mass loss and/or fat mass gain.^50^

The 60 studies included 112 cohorts: 40 cohorts contained participants who had undergone bariatric surgery, 33 cohorts contained participants who were receiving diet therapies (all except 2^65,84^ designed for weight loss or weight maintenance), 16 cohorts contained participants who received a medication or supplement, 7 cohorts contained participants who were following exercise regimens, 14 cohorts contained participants who were followed up for other reasons (ie, prostate cancer,^27^ kidney transplants,^78^ gene-associated obesity,^34^ diabetes vs prediabetes,^32^ polycystic ovary syndrome,^61^ and mindfulness intervention^81^), and 21 cohorts contained control participants (of which 4 cohorts contained participants who received placebo^31,35,51,66^). The size of the cohorts ranged from 5 to 335 participants (median, 32). The mean ages ranged from 18 to 71 years (median, 47 years). The percentage of men ranged from 0 to 100% (median, 35%).

The mean BMIs of the patients ranged from 23 to 54 (median, 38) (eTable 2 in the Supplement). Similarly, mean weight (median, 94 kg; range, 50-156 kg), fat mass (median, 32 kg; range, 20-47 kg), and percentage of body fat (median, 41%; range, 27%-53%) were high compared with general populations. Mean fasting insulin level (median, 13.53 μIU/mL; range, 4.32-27.79 μIU/mL [to convert to picomoles per liter, multiply by 6.945]), and the homeostatic model assessment index (median, 3.3; range, 0.9-12.9) were also high. Most of the mean CRP levels corresponded to a low-grade inflammation (median, 0.52 mg/dL; interquartile range, 0.21-0.75 mg/dL; range, 0.06-5.62 mg/dL [to convert to milligrams per liter, multiply by 10]). Mean interleukin 6 level ranged from 1.3 to 19.8 pg/mL (median, 3.4 pg/mL) and mean tumor necrosis factor α levels from 3.1 to 19.2 pg/mL (median, 12.4 pg/mL).

### Risk of Bias Assessment

**Figure 2.**
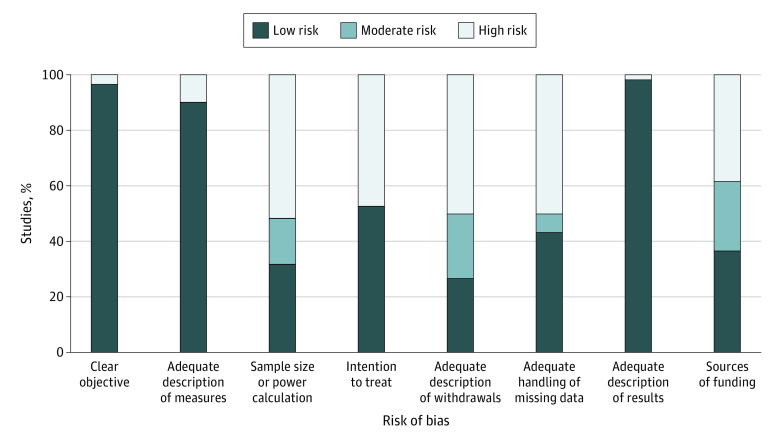
Risk of Bias Assessment Except for 3 items (clear objective, adequate description of measures, and adequate description of results), the assessments indicate several risks of bias.

Studies were largely rated as low risk for description of the objectives (96.7%), the outcome measures (90.0%), and the results (98.3%) (Figure 2). Approximately half the studies were high risk because they lacked a sample size or power calculation (51.7%), they (in those studies that assigned the interventions) did not take an intention-to-treat approach (47.2%), they had a withdrawal rate greater than 20% or they did not adequately describe their withdrawals (50.0%), or they did not adequately explore the effect of missing data (50.0%). In addition, 38.3% of studies had an industry source of funding.

### BMI and Fasting Insulin Level

**Figure 3.**
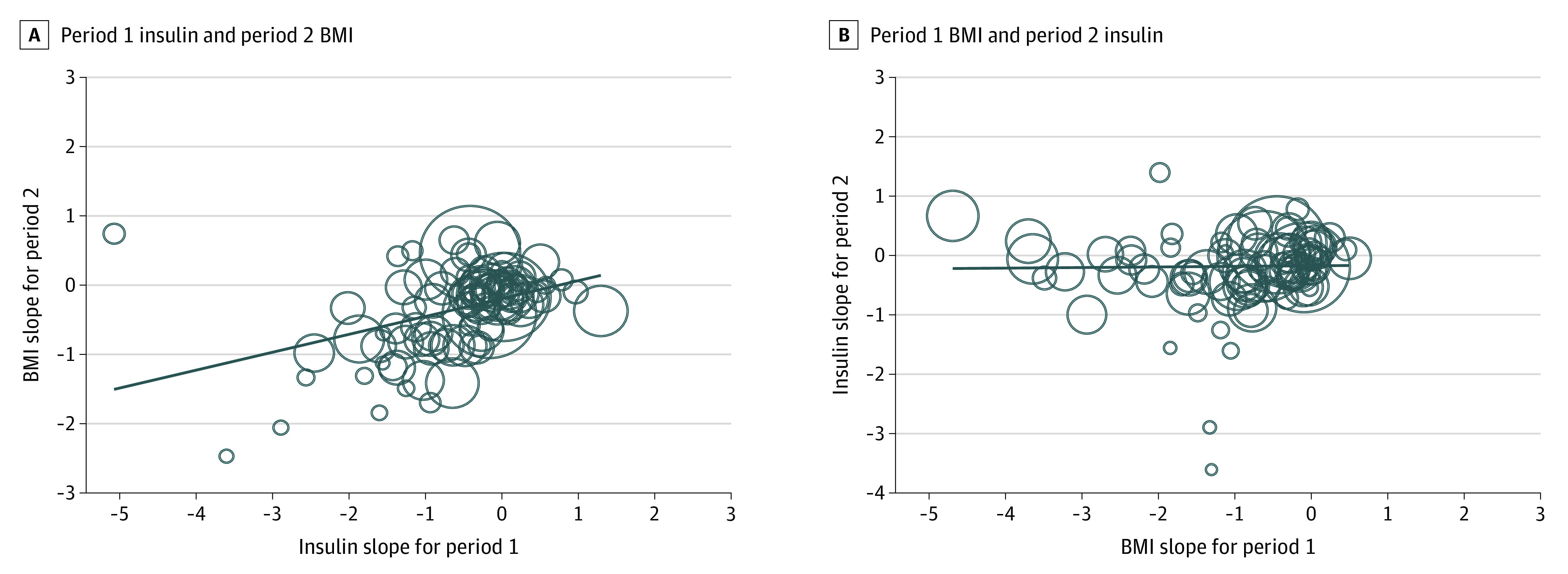
Bubble Plot of Temporal Associations Between Period 1 and Period 2 Changes A, Period 2 change in body mass index (BMI) (or standardized slope) is regressed onto period 1 change in insulin. B, Period 2 change in insulin is regressed onto period 1 change in BMI. The flat trend line in panel B suggests no association between period 1 change in BMI and period 2 change in insulin. The diagonal trend line in panel A supports a positive and temporal association between period 1 change in insulin and period 2 change in BMI. The size of the circles is based on the inverse of the SE of each cohort.

There were 90 pairs of standardized slopes from 56 cohorts and 35 studies that measured BMI and fasting insulin (Table 2). Most BMI and fasting insulin standardized slopes were negative (81% for BMI and 71% for fasting insulin), meaning that participants in most studies experienced decreases in BMI and insulin. The association between period 1 fasting insulin level and period 2 BMI was positive and significant (β = 0.26; 95% CI, 0.13-0.38; *I^2^* = 79%) (Figure 3), indicating that for every unit of SD change in period 1 insulin, there was an associated change in 0.26 units of SD in period 2 BMI. The association between period 1 BMI and period 2 fasting insulin level was not significant (β = 0.01; 95% CI, –0.08 to 0.10; *I*^2^ = 69%) (Figure 3). The heterogeneities were large.

**Table 2.** Pooled Temporal Associations: Primary Analysis^a^

Dependent (Period 2)	Independent (Period 1)	No. of cohorts	β (95% CI)	*I*^2^/τ^2^
**BMI and insulin**				
ΔBMI	ΔInsulin	90	0.26 (0.13 to 0.38)	79%/0.161
ΔInsulin	ΔBMI	90	0.01 (–0.08 to 0.10)	69%/0.099
**BMI and CRP**				
ΔBMI	ΔCRP	57	0.23 (–0.09 to 0.55)	83%/0.168
ΔCRP	ΔBMI	57	0.20 (0.04 to 0.36)	53%/0.048
**Insulin and CRP**				
ΔInsulin	ΔCRP	42	0.19 (–0.04 to 0.42)	49%/0.038
ΔCRP	ΔInsulin	42	0.29 (0.10 to 0.47)	36%/0.023

Abbreviations: BMI, body mass index (calculated as weight in kilograms divided by height in meters squared); CRP, C-reactive protein; Δ, change.

^a^ Each row describes one model where change in a measure, specifically a standardized slope, of a later period (period 2) is regressed on a change in a different measure of an earlier period (period 1).

The associations between insulin level and BMI increased in magnitude when studies that reported findings at 12 weeks or less were isolated from those that reported findings at greater than 12 weeks (eTable 3 in the Supplement). The magnitude of association between period 1 fasting insulin level and period 2 BMI was greater at 12 weeks or less than at greater than 12 weeks (β = 0.61; 95% CI, 0.38-0.84 vs β = 0.17; 95% CI, 0.05-0.30; *I*^2^ = 76%, *P* = .001). The association between period 1 fasting insulin level and period 2 BMI was present in participants who had undergone bariatric surgery but not in participants who had not undergone bariatric surgery (β = 0.31; 95% CI, 0.19-0.44 vs β = –0.12; 95% CI, –0.41 to 0.18; *I*^2^ = 76%, *P* = .007) (eTable 4 in the Supplement).

### BMI and CRP

There were 57 pairs of standardized slopes from 39 cohorts and 22 studies that measured both BMI and CRP levels (Table 2). Most standardized slopes for BMI and CRP were negative (81% for BMI and 68% for CRP), suggesting that participants in most studies experienced decreases in BMI and CRP level. The association between period 1 CRP level and period 2 BMI was not significant (β = 0.23; 95% CI, –0.09 to 0.55; *I*^2^ = 83%). The association between period 1 BMI and period 2 CRP level was positive and significant (β = 0.20; 95% CI, 0.04-0.36; *I*^2^ = 53%), suggesting that for every unit of SD change in period 1 BMI, there was an associated change of 0.20 units of SD in period 2 CRP level. However, both β coefficients were positive and had similar magnitudes, and the β coefficient for BMI had larger heterogeneity.

The associations between BMI and CRP level increased in magnitude when the studies that reported findings at 12 weeks or less were isolated from those that reported findings at greater than 12 weeks, when period 2 BMI was regressed on period 1 CRP level (eTable 3 in the Supplement). Although not significantly so, the magnitude of the association between period 1 CRP level and period 2 BMI was greater at 12 weeks or less than at greater than 12 weeks (β = 0.72; 95% CI, 0.08-1.37 vs β = 0.14; 95% CI, –0.18 to 0.47; *I*^2^ = 81%, *P* = .09). In addition, the association between period 1 CRP level and period 2 BMI was present in participants who underwent bariatric surgery but not in participants who had not undergone bariatric surgery (β = 0.43; 95% CI, 0.10-0.76 vs β = –0.40; 95% CI, –0.93 to 0.13; *I*^2^ = 81%, *P* = .005) (eTable 4 in the Supplement).

### Fasting Insulin and CRP

There were 42 pairs of standardized slopes from 27 cohorts and 16 studies that measured both fasting insulin and CRP levels (Table 2). Most fasting insulin and CRP standardized slopes were negative (74% of fasting insulin slopes and 63% of CRP slopes), suggesting that participants in most studies experienced decreases in insulin and CRP levels. The association between period 1 CRP level and period 2 fasting insulin level was not significant (β = 0.19; 95% CI, –0.04 to 0.42; *I*^2^ = 49%). The association between period 1 fasting insulin level and period 2 CRP level was positive and significant (β = 0.29; 95% CI, 0.10-0.47; *I*^2^ = 36%), suggesting that for every unit of SD change in period 1 insulin level, there was an associated change of 0.29 units of SD in period 2 CRP level. There was moderate heterogeneity. The subgroups did not significantly modify the associations between fasting insulin and CRP levels (eTables 2 and 3 in the Supplement).

### Other Sensitivity Analyses

When we considered related measures of BMI (weight, fat mass, and fat percentage), homeostatic model assessment index, and the other inflammatory markers (ie, interleukin 6 and tumor necrosis factor α), the associations among these variables were similar to those for BMI or could not be calculated (eTable 5 in the Supplement). The results when adjusting for nonindependence when available were similar (eTable 6 in the Supplement)—1 of the 6 models did not converge likely because of overly identified models (too few data for the number of model parameters). When we considered 2 measures as independent variables, the association of period 1 insulin level on period 2 BMI remained significant when the period 1 CRP level remained in the model (β = 0.57; 95% CI, 0.27-0.86 and β = –0.07; 95% CI, –0.42 to 0.29; *I*^2^ = 81%) (eTable 7 in the Supplement).

## Discussion

This systematic review and meta-analysis suggests that decreases in fasting insulin are more likely to precede decreasing weight than are decreases in weight to precede decreasing levels in fasting insulin. After accounting for the association between preceding levels of fasting insulin and the subsequent likelihood of weight gain, there was no evidence that inflammation preceded subsequent weight gain (eTable 7 in the Supplement). This temporal sequencing (in which changes in fasting insulin precede changes in weight) is not consistent with the assertion that obesity causes NCDs and premature death by increasing levels of fasting insulin.

### Support From Other Studies

In patients with type 2 diabetes, RCTs have found that introducing exogenous insulin and sulfonylureas (which increase endogenous insulin production) compared with lower doses or no drug therapy produce increases in weight.^85,86^ Some patients with type 1 diabetes deliberately omit or reduce their insulin injections to lose weight.^87^ Similarly, reports after bariatric surgery consistently indicate that insulin levels decrease before weight decreases in patients undergoing bariatric surgery.^88^ Thus, the finding that changes in insulin levels tend to precede changes in weight rather than the other way around has been previously demonstrated in 3 different scenarios. To our knowledge, there is no clinical evidence demonstrating that weight gain or loss precedes increases or decreases in endogenous insulin.

### Importance of the Findings

Obesity as a cause of premature death fails to meet several of the Bradford Hill criteria for causation: the strength of association is small^3^; the consistency of effect across older and/or ill populations favors obesity^4,5,6,7,8,9,10,11,12,13,14,15,16^; and the biological gradient is U-shaped, with overweight and obesity level 1 associated with the lowest risk^3^; and if hyperinsulinemia is to be considered the mediator, then the temporal sequencing is incorrect.

Insulin resistance, a cause and consequence of hyperinsulinemia,^89^ leads to type 2 diabetes and is associated with other adverse outcomes, such as myocardial infarction, chronic pulmonary disease, and some cancers,^90,91^ and may also be indicated in diabetic nephropathy.^92^ Despite the 3 scenarios described earlier, it is commonly believed that obesity leads to hyperinsulinemia.^93,94,95^ If the converse is true and hyperinsulinemia actually leads to obesity and its putative adverse consequences, then weight loss without concomitant decreases in insulin (eg, liposuction) would not be expected to address these adverse consequences. In addition, weight loss would not address risk in people with so-called metabolically healthy obesity, that is, those without insulin resistance.^96^

Of interest, insulin resistance is also present in lean individuals, in particular men and individuals of Asian descent.^97^ These 2 groups are at heightened risk for type 2 diabetes^98^ and cardiovascular disease, yet are more likely to be lean than women and individuals not of Asian descent. These observations are consistent with the hypothesis that hyperinsulinemia rather than obesity is driving adverse outcomes in this population. We speculate that the capacity to store the byproducts of excess glucose by increasing the size of fat cells (manifested as obesity) might delay the onset of type 2 diabetes and its consequences in some individuals, thus explaining the so-called obesity paradox of lower mortality among people with obesity. This idea, although not new,^99^ fits better with the emerging evidence. If this speculation is correct, assessing the capacity to store such by-products at the individual level may be a useful step toward personalized medicine.

Although it is possible that hyperinsulinemia per se is not the causal agent that leads to adverse outcomes (but is rather a marker for another more proximate factor), this would not change the lack of support for recommending weight loss among people with obesity. Rather, other markers should be investigated that, although correlated with obesity, are more strongly associated with premature mortality because they also exist in lean individuals. Therapies that lower insulin levels (eg, moderate diets with fewer simple carbohydrates and metformin) may be sustainable if an intermediate marker other than weight is targeted. Because the prevalence of obesity continues to increase worldwide, additional studies to confirm this hypothesis are urgently needed, not least because public health campaigns promoting weight loss are ineffective and lead to stigma^100^ among those with obesity.

### Limitations

This study has limitations. First, the identified studies largely enrolled participants with chronic obesity undergoing weight loss interventions and measures of interest (eg, weight, insulin level, and CRP level) mostly decreased. The findings are limited to those individuals losing weight and, given the findings from the bariatric subgroup analysis, are likely driven by quick decreases in circulating insulin levels (eTable 4 in the Supplement). Second, the included populations mostly had baseline mean CRP levels between 1 and 10 mg/L (eTable 2 in the Supplement), suggesting a low grade of chronic inflammation normally associated with atherosclerosis and insulin resistance. A number of studies^90,101,102,103,104^ have highlighted a group of people characterized by CRP levels consistently greater than 10 mg/L. Although this higher grade of chronic inflammation is associated with obesity, few participants had insulin resistance, suggesting a distinct grouping.^90^ Third, this meta-analysis used summary-level rather than individual patient–level data and is therefore vulnerable to the ecologic fallacy. A prospective cohort study designed for weight loss or gain with very frequent measurements in a diverse population would contribute a stronger form of evidence. Fourth, the review was limited to studies published in 2018, and many of the studies indicate a significant risk of bias with respect to their stated goals. However, none of the studies were designed to measure temporal associations between the measures of interest, so these limitations in study conduct would not necessarily have led to bias with respect to the findings. Although the search was limited to a single publication year (2018) to reduce the workload associated with this review, there is no reason to expect that data from this year would differ from data published earlier or later.

## Conclusions

The pooled evidence from this meta-analysis suggests that decreases in fasting insulin levels precede weight loss; it does not suggest that weight loss precedes decreases in fasting insulin. This temporal sequencing is not consistent with the assertion that obesity causes NCDs and premature death by increasing levels of fasting insulin. This finding, together with the obesity paradox, suggests that hyperinsulinemia or another proximate factor may cause the adverse consequences currently attributed to obesity. Additional studies to confirm this hypothesis are urgently needed.

## Notes

Supplement. **eTable 1.** Search Strategies **eTable 2.** Study Population Characteristics **eTable 3.** Pooled Temporal Associations: Subgroup Group Analysis ≤12 vs >12 Weeks **eTable 4.** Pooled Temporal Associations: Subgroup Group Analysis Bariatric vs Non-bariatric Patients **eTable 5.** Pooled Temporal Associations: Extended Analysis (All Measures) **eTable 6.** Pooled Temporal Associations: Sensitivity Analysis Adjusting for Non-independence **eTable 7.** Pooled Temporal Associations: Sensitivity Analysis Adjusting for Two Independent Variables
