# Supplementary material for: Temporal Associations Among Body Mass Index, Fasting Insulin, and Systemic Inflammation: A Systematic Review and Meta-analysis
Source: JAMA Netw Open. 2021 Mar 12;4(3):e211263. doi: 10.1001/jamanetworkopen.2021.1263 (PMC7955272; doi:10.1001/jamanetworkopen.2021.1263)
Supplement: Supplement. — eTable 1. Search Strategies eTable 2. Study Population Characteristics eTable 3. Pooled Temporal Associations: Subgroup Group Analysis ≤12 vs >12 Weeks eTable 4. Pooled Temporal Associations: Subgroup Group Analysis Bariatric vs Non-bariatric Patients eTable 5. Pooled Temporal Associations: Extended Analysis (All Measures) eTable 6. Pooled Temporal Associations: Sensitivity Analysis Adjusting for Non-independence eTable 7. Pooled Temporal Associations: Sensitivity Analysis Adjusting for Two Independent Variables [file jamanetwopen-e211263-s001.pdf]

Supplementary Online Content

Wiebe N, Ye F, Crumley ET, Bello A, Stenvinkel P, Tonelli M. Temporal associations among body mass index, fasting insulin, and systemic inflammation: a systematic review and meta-analysis. *JAMA Netw Open*. 2021;4(3):e211263. doi:10.1001/jamanetworkopen.2021.1263

eTable 1. Search Strategies
eTable 2. Study Population Characteristics
eTable 3. Pooled Temporal Associations: Subgroup Group Analysis ≤12 vs >12 Weeks
eTable 4. Pooled Temporal Associations: Subgroup Group Analysis Bariatric vs Non-bariatric Patients
eTable 5. Pooled Temporal Associations: Extended Analysis (All Measures)
eTable 6. Pooled Temporal Associations: Sensitivity Analysis Adjusting for Non-independence
eTable 7. Pooled Temporal Associations: Sensitivity Analysis Adjusting for Two Independent Variables

This supplementary material has been provided by the authors to give readers additional information about their work.

© 2021 Wiebe N et al. *JAMA Network Open.*

eTable 1. Search Strategies

Database	Search Strategies	Date and Coverage
MEDLINE	1. C-Reactive Protein/<br>2. CRP.ti,ab.<br>3. "C-reactive protein".ti,ab.<br>4. (fasting adj3 insulin).ti,ab.<br>5. HOMA*.ti,ab.<br>6. "homeostatic model assessment of insulin resistance".ti,ab.<br>7. "homeostasis model assessment of insulin resistance".ti,ab.<br>8. exp interleukins/<br>9. interleukin*.ti,ab.<br>10. QUIKI*.ti,ab.<br>11. QUICKI*.ti,ab.<br>12. "quantitative insulin sensitivity check index".ti,ab.<br>13. Tumor Necrosis Factor-alpha/<br>14. mhr24.ti,ab.<br>15. "tumour necrosis factor*".ti,ab.<br>16. "tissue necrosis factor*".ti,ab.<br>17. "tumor necrosis serum*".ti,ab.<br>18. "tumour necrosis serum*".ti,ab.<br>19. TNFalpha.ti,ab.<br>20. TNF-alpha.ti,ab.<br>21. TNF alfa.ti,ab.<br>22. "tumor necrosis factor*".ti,ab.<br>23. Cachectin.ti,ab.<br>24. Cachetin.ti,ab.<br>25. or/1-24<br>26. body fat distribution/<br>27. Body fat meter.ti,ab.<br>28. "fat distribut*".ti,ab.<br>29. body mass index/<br>30. BMI.ti,ab.<br>31. "body mass index".ti,ab.<br>32. "Quetelet* Index".ti,ab.<br>33. ("fat mass" or "percentage body fat" or Skin fold thickness or "weight change*" or "weight gain" or "weight loss" or Waist circumference or "Waist circumference" or "waist size" or "skinfold measure*").ti,ab.<br>34. Waist-Hip Ratio/ or exp body weight changes/ or Body-Weight Trajectory/ or weight gain/ or weight loss/ or waist circumference/ or Skinfold thickness/<br>35. (("waist to hip" or waist-hip or "hip to waist" or hip-waist) adj3 ratio).ti,ab.<br>36. (weight adj3 trajectory).ti,ab.<br>37. (weight adj3 reduc*).ti,ab.<br>38. or/26-37<br>39. 25 and 38<br>40. "randomized controlled trial".pt.<br>41. (random$ or placebo$ or single blind$ or double blind$ or triple blind$).ti,ab.	Ovid MEDLINE(R) and Epub Ahead of Print, In-Process & Other Non-Indexed Citations and Daily 1946 to August 20, 2019

© 2021 Wiebe N et al. *JAMA Network Open.*

Database	Search Strategies	Date and Coverage
	42. (retraction of publication or retracted publication).pt.<br><br>43. or/40-42<br>44. (animals not humans).sh.<br>45. ((comment or editorial or meta-analysis or practice-guideline or review or letter) not "randomized controlled trial").pt.<br>46. (random sampl$ or random digit$ or random effect$ or random survey or random regression).ti,ab. not "randomized controlled trial".pt.<br>47. 43 not (44 or 45 or 46)<br>48. controlled clinical trial.pt.<br>49. epidemiologic methods/<br>50. epidemiologic studies/<br>51. exp Case-Control Studies/<br>52. (epidemiologic adj (study or studies)).ab,ti.<br>53. case control.ab,ti.<br>54. (cohort adj (study or studies)).ab,ti.<br>55. cohort analy$.ab,ti.<br>56. (follow up adj (study or studies)).ab,ti.<br>57. (observ$ adj3 (study or studies)).ab,ti.<br>58. or/48-57<br>59. 47 or 58<br>60. exp Cross-Sectional Studies/<br>61. 59 not 60<br>62. 39 and 61<br>63. (change or reduc* or decreas* or increas* or loss or gain or trajectory or pre-post).mp.<br>64. 62 and 63<br>65. limit 64 to (english language and humans)<br>66. limit 65 to year=”2018”	
EMBASE<br>404-2128	1. C-Reactive Protein.ti,ab.<br>2. exp C reactive protein/<br>3. CRP.ti,ab.<br>4. exp insulin blood level/<br>5. (fasting adj3 insulin).ti,ab.<br>6. exp homeostasis model assessment/<br>7. homa*.ti,ab.<br>8. homeostatic model assessment of insulin resistance.ti,ab.<br><br>9. "homeostasis model assessment of insulin resistance".ti,ab.<br>10. exp interleukin derivative/<br>11. interleukin*.ti,ab.<br>12. QUIKI*.ti,ab.<br>13. exp Quantitative Insulin Sensitivity Check Index/<br>14. quicki.ti,ab.<br>15. "quantitative insulin sensitivity check index".ti,ab.<br><br>16. exp tumor necrosis factor/<br>17. Tumor Necrosis Factor-alpha.ti,ab.<br>18. TNF-alpha.ti,ab.<br>19. TNF alfa.ti,ab.<br>20. TNFalpha.ti,ab.<br>21. TNFalfa.ti,ab.<br>22. tissue necrosis factor*.ti,ab.	Embase 1974 to August 19, 2019

© 2021 Wiebe N et al. *JAMA Network Open.*

Database	Search Strategies	Date and Coverage
	23. "tumor necrosis factor*".ti,ab.<br>24. "tumour necrosis factor*".ti,ab.<br>25. "tumor necrosis serum*".ti,ab.<br>26. "tumour necrosis serum*".ti,ab.<br>27. mhr24.ti,ab.<br>28. Cachectin.ti,ab.<br>29. cachetin.ti,ab.<br>30. or/1-29<br>31. exp body fat distribution/<br>32. exp body fat meter/<br>33. Body fat meter.ti,ab.<br>34. "fat distribut*".ti,ab.<br>35. exp body mass/<br>36. BMI.ti,ab.<br>37. "body mass index".ti,ab.<br>38. Body ban mass.ti,ab.<br>39. "Quetelet* Index".ti,ab.<br>40. ("fat mass" or "percentage body fat" or Skin fold thickness or "weight change*" or "weight gain" or "weight loss" or Waist circumference or "Waist circumference" or "waist size" or "skinfold measure*").ti,ab.<br>41. Waist-Hip Ratio/ or exp body weight change/ or body weight gain/ or body weight loss/ or waist circumference/ or Skinfold thickness/<br>42. exp waist hip ratio/<br>43. (("waist to hip" or waist-hip or "hip to waist" or hip-waist) adj3 ratio).ti,ab.<br>44. (weight adj3 trajectory).ti,ab.<br>45. (weight adj3 reduc*).ti,ab.<br>46. or/31-45<br>47. 30 and 46<br>48. (random$ or placebo$ or single blind$ or double blind$ or triple blind$).ti,ab.<br>49. RETRACTED ARTICLE/<br>50. or/48-49<br>51. (animal$ not human$).sh,hw.<br>52. (book or conference paper or editorial or letter or review).pt. not exp randomized controlled trial/<br>53. (random sampl$ or random digit$ or random effect$ or random survey or random regression).ti,ab. not exp randomized controlled trial/<br>54. 50 not (51 or 52 or 53)<br>55. exp cohort analysis/<br>56. exp longitudinal study/<br>57. exp prospective study/<br>58. exp follow up/<br>59. cohort$.tw.<br>60. or/55-59<br>61. 54 or 60<br>62. 47 and 61<br>63. (change or reduc* or decreas* or increas* or loss or gain or trajectory or pre-post).ti,ab.<br>64. 62 and 63<br>65. exp Cross-Sectional Studies/<br>66. 64 not 65	

© 2021 Wiebe N et al. *JAMA Network Open.*

Database	Search Strategies	Date and Coverage
	67. limit 66 to (human and english language)<br>68. limit 67 to year="2018"	

© 2021 Wiebe N et al. *JAMA Network Open.*

eTable 2. Study Population Characteristics

Study	Cohort(s)	N	Mean BMI, kg/m^2	Mean weight, kg	Mean fat, kg	Mean fat, %	Mean insulin, pmol/L	Mean HOMA	Mean CRP, mg/L	Mean IL-6, pg/mL	Mean TNF-ɒ, pg/mL
Abdel-Razik et al,[35] 2018	Rifaximin	25	33	.	.	.	83	3.9	.	8.3	19.2
	Placebo	25	33	.	.	.	82	3.7	.	8.1	19.0
Abiad et al,[37] 2018	PCOS	6	41	111	.	.	144	.	6.6	.	.
	Control	16	41	107	.	.	111	.	10.0	.	.
Arikawa et al,[82] 2018	CR diet plus exercise	10	.	86	.	.	54	1.8	6.0	2.7	.
	Weight management counselling	10	.	98	.	.	116	5.4	5.2	2.8	.
Arnold et al,[72] 2018	Decreased added sugars, increased fiber and fish diet	14	39	103	.	.	96	.	7.4	1.3	.
Asle Mohammadi Zadeh et al,[55] 2018	Low-carbohydrate diet	11	.	100	.	.	130	2.5	.	2.4	3.4
	Low-fat diet	11	.	108	.	.	131	2.5	.	2.4	3.1
	High-fat diet	11	.	105	.	.	137	2.6	.	2.6	3.2
	Control	9	.	100	.	.	130	2.5	.	2.1	3.5
Baltieri et al,[83] 2018	RYGB	13	36	96	.	.	.	.	8.0	19.8	15.8
Bulatova et al,[46] 2018	Metformin	26	37	.	.	.	.	.	.	.	.
	Control	27	40	.	.	.	.	.	.	.	.
Carbone et al,[67] 2019[a]	T2D remission	14	39	108	.	.	97	2.3	.	.	.
	No T2D remission	27	31	93	.	.	46	1.4	.	.	.
Chen et al,[29] 2018	Saxagliptin and metformin	51	24	.	.	.	.	2.6	.	.	.
	Acarbose and metformin	51	24	.	.	.	.	2.6	.	.	.
Cheung et al,[42] 2018	Cessation of androgen deprivation therapy	27	.	.	27	.	.	2.3	.	.	.
	Control	19	.	.	27	.	.	2.3	.	.	.
Chiappetta et al,[73] 2018	SG	241	53	156	.	.	.	.	11.7	.	.
	One-anastomosis GB	68	49	144	.	.	.	.	10.6	.	.
	RYGB	159	45	128	.	.	.	.	8.6	.	.
Dardzińska et al,[74] 2018	Mini-GB	9	42	.	.	.	65	2.5	.	.	.
	SG	5	42	.	.	.	63	2.4	.	.	.

© 2021 Wiebe N et al. *JAMA Network Open.*

Study	Cohort(s)	N	Mean BMI, kg/m^2	Mean weight, kg	Mean fat, kg	Mean fat, %	Mean insulin, pmol/L	Mean HOMA	Mean CRP, mg/L	Mean IL-6, pg/mL	Mean TNF-ɒ, pg/mL
	RYGB	9	45	.	.	.	73	2.7	.	.	.
De Luis, Calvo et al,[34] 2018	CC[c] rs266729	46	46	123	47	.	103	4.2	.	.	.
	CG or GG rs266729	84	49	124	43	.	115	4.3	.	.	.
De Luis, Izaola et al,[49] 2018	Mediterranean CR diet then dietary counseling	335	35	92	37	.	81	3.3	5.2	.	.
De Luis, Pacheco et al,[64] 2018	GG[c] rs670	17	47	128	45	.	101	4.1	.	.	.
	GA or AA rs670	65	49	124	44	.	108	4.1	.	.	.
De Paulo et al,[28] 2018	Aerobic and resistance training	18	.	67	31	46	.	.	5.0	.	.
	Stretching and relaxation exercises	18	.	72	34	46	.	.	7.0	.	.
Demerdash et al,[76] 2018	SG	92	47	130	.	.	51	2.6	.	.	.
Derosa et al,[40] 2018	Canrenone	92	28	82	.	.	103	5.5	.	.	.
	Hydrochlorothiazide	90	28	80	.	.	109	5.6	.	.	.
Dhillon et al,[84] 2018	Almond snacks	38	.	72	20	27	34	1.4	.	.	.
	Cracker snacks	35	.	71	20	28	40	1.8	.	.	.
Di Sebastiano et al,[27] 2018	Treated	8	28	86	27	31	88	3.3	2.3	11.9	12.4
Drummen et al,[59] 2018 (PREVIEW)	High-protein diet	12	31	92	.	41	71	3.2	.	.	.
	Moderate-protein diet	13	31	94	.	41	89	4.1	.	.	.
Esquivel et al,[69] 2018	SG	63	45	129	.	.	94	3.9	.	.	.
Fortin et al,[54] 2018	Mediterranean diet	14	32	91	.	39	.	.	2.4	.	.
	Low-fat diet	14	30	88	.	36	.	.	2.1	.	.
	High-egg diet	66	.	97	.	30	.	.	4.8	3.4	.

© 2021 Wiebe N et al. *JAMA Network Open.*

Study	Cohort(s)	N	Mean BMI, kg/m^2	Mean weight, kg	Mean fat, kg	Mean fat, %	Mean insulin, pmol/L	Mean HOMA	Mean CRP, mg/L	Mean IL-6, pg/mL	Mean TNF-ɒ, pg/mL
Fuller et al,[26] 2018 (DIABEGG)	Low-egg diet	62	.	91	.	30	.	.	4.4	2.9	.
Gadéa et al,[56] 2018	Chemotherapy	38-50[b]	.	69	26	.	.	1.7	4.5	.	.
Galbreath et al,[62] 2018	High-protein diet	17	.	82	33	44	76	3.7	.	.	.
	High-carbohydrate diet	18	.	79	32	44	61	2.5	.	.	.
	Control	19	.	76	30	43	67	3.2	.	.	.
Goday et al,[77] 2018	Helicobacter pylori eradication before SG	49	43	.	.	.	.	6.9	.	.	.
	Control before SG	60	43	.	.	.	.	6.2	.	.	.
	Helicobacter pylori eradication before RYGB	50	43	.	.	.	.	5.6	.	.	.
	Control before RYGB	70	43	.	.	.	.	6.3	.	.	.
Guarnotta et al,[36] 2018	Pasireotide	12	36	98	.	.	46	0.9	.	.	.
Hady et al,[45] 2018	32 French bougie size in SG	40	50	141	.	.	193	9.6	9.5	.	.
	36 French bougie size in SG	40	48	136	.	.	135	6.5	9.6	.	.
	40 French bougie size in SG	40	45	131	.	.	126	5.6	7.5	.	.
Hanai et al,[44] 2018	EPA-enriched nutritional supplement	13	.	50	.	.	.	.	1.3	.	.
	Control	14	.	51	.	.	.	.	0.7	.	.
Hattori et al,[66] 2018	Empagliflozin	51	31	.	.	.	65	2.6	1.3	.	.
	Placebo	51	30	.	.	.	55	3.6	1.5	.	.
Kazemi et al,[61] 2018	Low-glycemic index pulse-based diet	16	30	82	32	.	81	.	3.2	.	.
	Therapeutic lifestyle change diet	16	35	96	43	.	88	.	5.2	.	.
Keinänen et al,[53] 2018	Treated	84-94[b]	23	.	.	.	53	1.6	0.7	.	.

© 2021 Wiebe N et al. *JAMA Network Open.*

Study	Cohort(s)	N	Mean BMI, kg/m^2	Mean weight, kg	Mean fat, kg	Mean fat, %	Mean insulin, pmol/L	Mean HOMA	Mean CRP, mg/L	Mean IL-6, pg/mL	Mean TNF-ɒ, pg/mL
Krishnan et al,[25] 2018	2010 American dietary guidelines diet	28	32	90	.	.	103	4.2	.	.	.
	Typical American diet	24	33	90	.	.	88	3.7	.	.	.
Lambert et al,[79] 2018	RYGB and BPD	109	39	102	41	39	119	5.2	7.7	.	.
Lee et al,[38] 2018	Bariatric surgery	44	.	117	.	.	.	.	.	.	.
	Control	25-44[b]	44	92	.	.	.	5.5	.	.	.
Liang et al,[52] 2018	Low-calorie diet	18	33	95	.	.	119	5.2	2.3	.	.
Liaskos et al,[70] 2018	SG	43	50	.	.	.	158	8.1	.	.	.
	RYGB	28	47	.	.	.	176	6.7	.	.	.
Liu et al,[60] 2018	RYGB	45	33	.	.	.	116	8.1	.	.	.
Madjd et al,[33] 2018	Diet beverages	36	34	88	.	.	79	3.1	.	.	.
	Water	35	34	88	.	.	82	3.2	.	.	.
Most et al,[41] 2018 (CALERIE 2)	25% CR	34	.	72	25	.	.	1.1	.	.	.
	Control	19	.	71	23	.	.	1.3	.	.	.
Mravić et al,[80] 2018	20% CR diet	30	29	83	.	40	.	.	4.8	.	.
	50% CR diet	30	31	86	.	41	.	.	5.8	.	.
	Alternating 70% and 30% CR diet	37	30	85	.	41	.	.	6.2	.	.
Munukka et al,[71] 2018	American College of Sports Medicine exercise program	17	32	90	39	44	55	12.9	.	.	.
Nicoletto et al,[78] 2018	Kidney transplantation	46	27	.	.	.	46	1.7	4.4	4.5	.
Nilholm et al,[30] 2018	Okinawan-based Nordic diet	30	30	.	.	.	93	.	.	.	14.9
Nishino et al,[39] 2018	Daikenchuto (TJ-100)	19	.	63	.	.	.	.	1.0	.	.
	Control	20	.	60	.	.	.	.	0.6	.	.

© 2021 Wiebe N et al. *JAMA Network Open.*

Study	Cohort(s)	N	Mean BMI, kg/m^2	Mean weight, kg	Mean fat, kg	Mean fat, %	Mean insulin, pmol/L	Mean HOMA	Mean CRP, mg/L	Mean IL-6, pg/mL	Mean TNF-ɒ, pg/mL
Patel et al,[57] 2018	Duodenal-jejunal sleeve bypass	41-45[b]	40	116	.	.	113	.	.	.	.
Raja Khan et al,[81] 2018	Mindfulness-based stress reduction	42	39	104	.	.	30	3.7	9.7	.	.
	Health education	44	39	102	.	.	32	4.0	10.7	.	.
Rubio Almanza et al,[32] 2018	Prediabetes	57	50	126	.	52	158	6.7	.	.	.
	T2D	48	54	132	.	53	132	8.5	.	.	.
Schübel et al,[58] 2018 (HELENA)	5:2 intermittent CR diet	49	.	96	.	.	.	2.7	4.2	.	.
	Continuous CR diet	49	.	93	.	.	.	3.0	4.1	.	.
	Control	52	.	93	.	.	.	3.0	5.4	.	.
Shah et al,[65] 2018 (EVADE CAD)	Vegan diet	50	31	.	.	.	65	.	1.3	.	.
	AHA diet	50	31	.	.	.	72	.	1.1	.	.
Sherf-Dagan et al,[31] 2018	Probiotic	40	42	.	.	.	.	6.5	11.5	.	.
	Placebo	40	42	.	.	.	.	5.6	12.3	.	.
Stolberg et al,[48] 2018	Moderate-intensity physical training	32-60[b]	43	129	.	.	173	.	6.1	3.8	.
	Control	28	43	124	.	.	143	.	6.1	3.8	.
Van Dammen et al,[43] 2018	Lifestyle intervention	289	36	104	.	.	97	3.3	5.6	.	.
	Control	285	36	103	.	.	104	3.6	5.6	.	.
Van Rijn et al,[47] 2018	Duodenal-jejunal bypass liner	28	37	113	.	.	129	.	.	.	.
Wilson et al,[50] 2018	Treated for TB	265-270[b]	.	57	.	.	.	.	56.2	.	.
	Control	82	.	60	.	.	.	.	3.9	.	.
Witczak et al,[63] 2018	Bariatric surgery	20	54	151	.	.	112	2.4	.	9.5	.
	HIIT	12	39	.	.	.	.	.	1.8	.	.

© 2021 Wiebe N et al. *JAMA Network Open.*

Study	Cohort(s)	N	Mean BMI, kg/m^2	Mean weight, kg	Mean fat, kg	Mean fat, %	Mean insulin, pmol/L	Mean HOMA	Mean CRP, mg/L	Mean IL-6, pg/mL	Mean TNF-ɒ, pg/mL
Wormgoor et al,[75] 2018	Moderate-intensity continuous training	11	35	.	.	.	.	.	2.1	.	.
Yang et al,[68] 2018	SG	10	38	.	.	.	132	.	.	.	.
	RYGB	10	39	.	.	.	127	.	.	.	.
Zhang et al,[51] 2018	Coenzyme Q10	51	25	63	.	.	57	2.5	1.1	.	.
	Placebo	50	25	65	.	.	66	3.0	1.0	.	.

AHA American Heart Association, BMI body mass index, BPD biliopancreatic diversion surgery, CR calorie restriction, CRP C-reactive protein, DM diabetes mellitus, GB Gastric Bypass, HOMA homeostatic model assessment, HIIT high-intensity interval training, IGT intolerance glucose test, IL-6 interleukin-6, MetS metabolic syndrome, na non-applicable, NAFLD non-alcoholic fatty liver disease, NASH non-alcoholic steatohepatitis, PCOS polycystic ovary syndrome, RYGB roux-en-y gastric bypass, SG sleeve gastrectomy, SGLT2 sodium-glucose transport protein 2, T1D type 1 diabetes, T2D type 2 diabetes, TB tuberculosis, TNF-ɒ tumor necrosis factor-alpha, WC waist circumference

[a]This study was published online in 2018.

[b]The number of measurements varied.

[c]AA, CC, CG, GA, GG are alleles.

© 2021 Wiebe N et al. *JAMA Network Open.*

eTable 3. Pooled Temporal Associations: Subgroup Group Analysis ≤12 vs >12 Weeks

Dependent (Period 2)	Independent (Period 1)	Number of weeks	N	β (95% CI)	I^2/Tau^2 P interaction
ΔBMI	ΔInsulin	All	90	**0.26 (0.13,0.38)**	79/0.161
		≤12	33	**0.61 (0.38,0.84)**	76/0.133
		>12	57	**0.17 (0.05,0.30)**	**P=0.001**
ΔInsulin	ΔBMI	All	90	0.01 (-0.08,0.10)	69/0.099
		≤12	33	**0.56 (0.32,0.80)**	62/0.073
		>12	57	-0.00 (-0.08,0.08)	**P<0.001**
ΔBMI	ΔCRP	All	57	0.23 (-0.09,0.55)	83/0.168
		≤12	27	**0.72 (0.08,1.37)**	81/0.153
		>12	30	0.14 (-0.18,0.47)	P=0.09
ΔCRP	ΔBMI	All	57	**0.20 (0.04,0.36)**	53/0.048
		≤12	27	0.22 (-0.03,0.48)	53/0.050
		>12	30	**0.20 (0.03,0.36)**	P=0.82
ΔInsulin	ΔCRP	All	42	0.19 (-0.04,0.42)	49/0.038
		≤12	22	0.45 (-0.05,0.94)	48/0.036
		>12	20	0.14 (-0.10,0.38)	P=0.25
ΔCRP	ΔInsulin	All	42	**0.29 (0.10,0.47)**	36/0.023
		≤12	22	0.20 (-0.02,0.42)	34/0.018
		>12	20	**0.43 (0.16,0.70)**	P=0.14

β beta coefficient/association, BMI body mass index, CI confidence interval, CRP C-reactive protein, Δ delta/change

Each set of three rows describes two models. The first row describes one model and uses all available cohorts where change in a measure, specifically a standardized slope, of a later time period (Period 2) is regressed on a change in a different measure of an earlier time period (Period 1). The second and third row describe a second model with an additional interaction term for a subgroup. The results are presented as the number of cohorts (N), the association (β) with its 95% confidence interval and two measures of between-cohort heterogeneity, I^2 and Tau^2. The P-value for interaction indicates whether the subgrouping is significant or not. Results significant at P<0.05 are in boldface.

© 2021 Wiebe N et al. *JAMA Network Open.*

eTable 4. Pooled Temporal Associations: Subgroup Group Analysis Bariatric vs Non-bariatric Patients

Dependent (Period 2)	Independent (Period 1)	Bariatric or not	N	β (95% CI)	I^2/Tau^2 P interaction
ΔBMI	ΔInsulin	All	90	**0.26 (0.13,0.38)**	79/0.161
		Yes	52	**0.31 (0.19,0.44)**	76/0.145
		No	38	-0.12 (-0.41,0.18)	**P=0.007**
ΔInsulin	ΔBMI	All	90	0.01 (-0.08,0.10)	69/0.099
		Yes	52	0.01(-0.08,0.10)	68/0.099
		No	38	-0.18 (-0.50,0.14)	P=0.23
ΔBMI	ΔCRP	All	57	0.23 (-0.09,0.55)	83/0.168
		Yes	26	**0.43 (0.10,0.76)**	81/0.142
		No	31	-0.40 (-0.93,0.13)	**P=0.005**
ΔCRP	ΔBMI	All	57	**0.20 (0.04,0.36)**	53/0.048
		Yes	26	**0.20 (0.04,0.36)**	54/0.051
		No	31	0.16 (-0.27,0.59)	P=0.84
ΔInsulin	ΔCRP	All	42	0.19 (-0.04,0.42)	49/0.038
		Yes	15	0.22 (-0.03,0.46)	50/0.040
		No	27	0.07 (-0.42,0.56)	P=0.58
ΔCRP	ΔInsulin	All	42	**0.29 (0.10,0.47)**	36/0.023
		Yes	15	**0.26 (0.06,0.45)**	36/0.021
		No	27	**0.46 (0.05,0.87)**	P=0.34

β beta coefficient/association, BMI body mass index, CI confidence interval, CRP C-reactive protein, Δ delta/change

Each set of three rows describes two models. The first row describes one model and uses all available cohorts where change in a measure, specifically a standardized slope, of a later time period (Period 2) is regressed on a change in a different measure of an earlier time period (Period 1). The second and third row describe a second model with an additional interaction term for a subgroup. The results are presented as the number of cohorts (N), the association (β) with its 95% confidence interval and two measures of between-cohort heterogeneity, I^2 and Tau^2. The P-value for interaction indicates whether the subgrouping is significant or not. Results significant at $P<0.05$ are in boldface.

© 2021 Wiebe N et al. *JAMA Network Open.*

eTable 5. Pooled Temporal Associations: Extended Analysis (All Measures)

Dependent (Period 2)	Independent (Period 1)	N	β (95% CI)	I^2/Tau^2
ΔBMI	ΔInsulin	90	**0.26 (0.13,0.38)**	79/0.161
ΔInsulin	ΔBMI	90	0.01 (-0.08,0.10)	69/0.099
ΔBMI	ΔHOMA	88	**0.19 (0.03,0.34)**	84/0.202
ΔHOMA	ΔBMI	88	0.01 (-0.05,0.08)	74/0.097
ΔBMI	ΔCRP	57	0.23 (-0.09,0.55)	83/0.168
ΔCRP	ΔBMI	57	**0.20 (0.04,0.36)**	53/0.048
ΔBMI	ΔIL-6	13	0.20 (-0.17,0.57)	29/0.043
ΔIL-6	ΔBMI	13	0.19 (-0.10,0.48)	20/0.038
ΔBMI	ΔTNF-ɒ	9	-0.56 (-1.31,0.20)	0/0
ΔTNF-ɒ	ΔBMI	9	-0.04 (-1.56,1.48)	86/0.755
ΔWeight	ΔInsulin	91	**0.16 (0.05,0.27)**	74/0.118
ΔInsulin	ΔWeight	91	0.04 (-0.08,0.17)	69/0.132
ΔWeight	ΔHOMA	83	0.12 (-0.02,0.26)	79/0.152
ΔHOMA	ΔWeight	83	0.01 (-0.12,0.13)	74/0.136
ΔWeight	ΔCRP	70	0.09 (-0.08,0.27)	75/0.093
ΔCRP	ΔWeight	70	-0.01 (-0.30,0.27)	71/0.144
ΔWeight	ΔIL-6	30	Could not calculate numerical derivatives	-
ΔIL-6	ΔWeight	30	0.21 (-0.01,0.44)	0/0
ΔWeight	ΔTNF-ɒ	18	-0.01 (-0.56,0.53)	0/0
ΔTNF-ɒ	ΔWeight	18	-0.01 (-0.88,0.87)	36/0.109
ΔFat mass	ΔInsulin	21	0.17 (-0.09,0.43)	80/0.085
ΔInsulin	ΔFat mass	21	0.08 (-0.28,0.44)	76/0.150
ΔFat mass	ΔHOMA	22	0.16 (-0.12,0.45)	81/0.100
ΔHOMA	ΔFat mass	22	-0.05 (-0.25,0.16)	81/0.129
ΔFat mass	ΔCRP	12	0.66 (-0.08,1.39)	78/0.100
ΔCRP	ΔFat mass	12	0.36 (-0.65,1.38)	58/0.144
ΔFat mass	ΔIL-6	1	Insufficient observations	
ΔIL-6	ΔFat mass	1		
ΔFat mass	ΔTNF-ɒ	1	Insufficient observations	
ΔTNF-ɒ	ΔFat mass	1		
ΔFat percent	ΔInsulin	16	0.23 (-0.01,0.47)	61/0.079

© 2021 Wiebe N et al. *JAMA Network Open.*

**Dependent (Period 2)**	**Independent (Period 1)**	**N**	**β (95% CI)**	**I^2/Tau^2**
ΔInsulin	ΔFat percent	16	0.24 (-0.05,0.52)	72/0.160
ΔFat percent<br>ΔHOMA	ΔHOMA<br>ΔFat percent	16<br>16	**0.25 (0.02,0.48)**<br>0.19 (-0.11,0.50)	53/0.061<br>70/0.174
ΔFat percent<br>ΔCRP	ΔCRP<br>ΔFat percent	11<br>11	0.54 (-0.01,1.09)<br>0.37 (-1.13,1.87)	29/0.040<br>64/0.187
ΔFat percent<br>ΔIL-6	ΔIL-6<br>ΔFat percent	1<br>1	Insufficient observations	
ΔFat percent<br>ΔTNF-ɒ	ΔTNF-ɒ<br>ΔFat percent	1<br>1	Insufficient observations	
ΔInsulin<br>ΔCRP	ΔCRP<br>ΔInsulin	42<br>42	0.19 (-0.04,0.42)<br>**0.29 (0.10,0.47)**	49/0.038<br>36/0.023
ΔInsulin<br>ΔIL-6	ΔIL-6<br>ΔInsulin	32<br>32	0.15 (-0.26,0.56)<br>0.12 (-0.07,0.31)	49/0.093<br>0/0.010
ΔInsulin<br>ΔTNF-ɒ	ΔTNF-ɒ<br>ΔInsulin	22<br>22	0.03 (-0.91,0.97)<br>-0.02 (-0.29,0.26)	64/0.351<br>26/0.039
ΔHOMA<br>ΔCRP	ΔCRP<br>ΔHOMA	39<br>39	-0.05 (-0.37,0.28)<br>-0.02 (-0.25,0.22)	72/0.059<br>64/0.083
ΔHOMA<br>ΔIL-6	ΔIL-6<br>ΔHOMA	27<br>27	0.18 (-0.35,0.71)<br>0.15 (-0.16,0.46)	29/0.042<br>0/0.008
ΔHOMA<br>ΔTNF-ɒ	ΔTNF-ɒ<br>ΔHOMA	21<br>21	0.20 (-0.53,0.93)<br>0.16 (-0.25,0.58)	44/0.128<br>21/0.008

β beta coefficient/association, BMI body mass index, CI confidence interval, CRP C-reactive protein, Δ delta/change, HOMA homeostatic model assessment, IL-6 interleukin-6, TNF-ɒ tumor necrosis factor-alpha

Each row describes one model where change in a measure, specifically a standardized slope, of a later time period (Period 2) is regressed on a change in a different measure of an earlier time period (Period 1). The results are presented as the number of cohorts (N), the association (β) with its 95% confidence interval and two measures of between-cohort heterogeneity, I^2 and Tau^2. Results significant at $P<0.05$ are in boldface.

© 2021 Wiebe N et al. *JAMA Network Open.*

eTable 6. Pooled Temporal Associations: Sensitivity Analysis Adjusting for Non-independence

Dependent (Period 2)	Independent (Period 1)	N	β (95% CI)	I^2/Tau^2
ΔBMI	ΔInsulin	90	**0.28 (0.22,0.33)**	84/0.239
ΔInsulin	ΔBMI	90	-0.00 (-0.08,0.08)	69/0.102
ΔBMI	ΔCRP	57	**0.37 (0.22,0.52)**	84/0.198
ΔCRP	ΔBMI	57	**0.19 (0.04,0.34)**	58/0.053
ΔInsulin	ΔCRP	42	-	-
ΔCRP	ΔInsulin	42	**0.29 (0.16,0.43)**	44/0.031

β beta coefficient/association, BMI body mass index, CI confidence interval, CRP C-reactive protein, Δ delta/change

Each row describes one model where change in a measure, specifically a standardized slope, of a later time period (Period 2) is regressed on a change in a different measure of an earlier time period (Period 1). The results are presented as the number of cohorts (N), the association (β) with its 95% confidence interval and two measures of between-cohort heterogeneity, I^2 and Tau^2. These models nest cohorts within studies. One row has no results because the model did not converge. Results significant at P<0.05 are in boldface.

© 2021 Wiebe N et al. *JAMA Network Open.*

eTable 7. Pooled Temporal Associations: Sensitivity Analysis Adjusting for Two Independent Variables

Dependent (Period 2)	Independent (Period 1)	N	β (95% CI)	I^2/Tau^2
ΔBMI	ΔInsulin<br>ΔCRP	38	**0.57 (0.27,0.86)**<br>-0.07 (-0.42,0.29)	81/0.109
ΔInsulin	ΔBMI<br>ΔCRP	38	**0.27 (0.00,0.55)**<br>-0.08 (-0.43,0.28)	54/0.041
ΔCRP	ΔBMI<br>ΔInsulin	38	0.26 (-0.01,0.54)<br>0.07 (-0.22,0.37)	35/0.016

β beta coefficient/association, BMI body mass index, CI confidence interval, CRP C-reactive protein, Δ delta/change

Each row describes one model where change in a measure, specifically a standardized slope, of a later time period (Period 2) is regressed on changes in two different measures of an earlier time period (Period 1). The results are presented as the number of cohorts (N), the association (β) with its 95% confidence interval and two measures of between-cohort heterogeneity, I^2 and Tau^2. Results significant at $P<0.05$ are in boldface.

© 2021 Wiebe N et al. *JAMA Network Open.*
